# Pgc-1α Overexpression Downregulates Pitx3 and Increases Susceptibility to MPTP Toxicity Associated with Decreased Bdnf

**DOI:** 10.1371/journal.pone.0048925

**Published:** 2012-11-07

**Authors:** Joanne Clark, Jessica M. Silvaggi, Tomas Kiselak, Kangni Zheng, Elizabeth L. Clore, Ying Dai, Caroline E. Bass, David K. Simon

**Affiliations:** 1 Department of Neurology, Beth Israel Deaconess Medical Center, Harvard Medical School, Boston, Massachusetts, United States of America; 2 Dana-Farber Cancer Institute, Harvard Medical School, Boston, Massachusetts, United States of America; 3 Department of Pharmacology and Toxicology, School of Medicine and Biosciences, University at Buffalo, Buffalo, New York, United States of America; University of Nebraska Medical Center, United States of America

## Abstract

Multiple mechanisms likely contribute to neuronal death in Parkinson’s disease (PD), including mitochondrial dysfunction and oxidative stress. Peroxisome proliferator-activated receptor gamma co-activator-1 alpha (PGC-1α) positively regulates the expression of genes required for mitochondrial biogenesis and the cell’s antioxidant responses. Also, expression of PGC-1α-regulated genes is low in substantia nigra (SN) neurons in early PD. Thus upregulation of PGC-1α is a candidate neuroprotective strategy in PD. Here, an adeno-associated virus (AAV) was used to induce unilateral overexpression of *Pgc-1α,* or a control gene, in the SN of wild-type C57BL/6CR mice. Three weeks after AAV administration, mice were treated with saline or MPTP. Overexpression of *Pgc-1α* in the SN induced expression of target genes, but unexpectedly it also greatly reduced the expression of tyrosine hydroxylase (Th) and other markers of the dopaminergic phenotype with resultant severe loss of striatal dopamine. Reduced Th expression was associated with loss of Pitx3, a transcription factor that is critical for the development and maintenance of dopaminergic cells. Expression of the neurotrophic factor *Bdnf*, which also is regulated by Pitx3, similarly was reduced. Overexpression of *Pgc-1α* also led to increased sensitivity to MPTP-induced death of Th+ neurons. Pgc-1α overexpression alone, in the absence of MPTP treatment, did not lead to cell loss in the SN or to loss of dopaminergic terminals. These data demonstrate that overexpression of *Pgc-1α* results in dopamine depletion associated with lower levels of Pitx3 and enhances susceptibility to MPTP. These data may have ramifications for neuroprotective strategies targeting overexpression of PGC-1α in PD.

## Introduction

Parkinson’s Disease (PD) is a neurodegenerative disorder characterized in part by loss of dopaminergic (DA) neurons of the substantia nigra (SN). Compelling evidence indicates central roles for mitochondrial dysfunction and oxidative stress [1]–[3]. Mitochondrial complex I impairment increases production of reactive oxygen species (ROS), consistent with a rise in levels of oxidative damage observed in the SN in PD [4]. Even in the absence of mitochondrial dysfunction, ROS are a byproduct of normal cellular respiration that, in the absence of adequate anti-ROS defenses, can lead to oxidative damage. Therefore, defects in endogenous antioxidant mechanisms can lead to ROS overload and are implicated in neurodegenerative disorders [5].

This, in part, led to an interest in peroxisome-proliferator-activated receptor-gamma co-activator 1 alpha (PGC-1α) as a potential therapeutic target in PD. PGC-1α is a transcriptional co-activator that can be induced by oxidative stress and coordinates the expression of multiple antioxidant programs [6], [7]. Transcription is a tightly regulated process; therefore, as a transcriptional co-activator, PGC-1α is a tightly regulated protein. PGC-1α has a defined pattern of tissue expression and occurs at higher levels in tissues with high metabolic requirement such as brown fat, skeletal muscle, kidney, heart and brain [8]–[10]. PGC-1α expression increases in response to various stimuli, such as physical exercise in muscle and hypothermia in brown fat [11]–[13]. As in muscle, PGC-1α activity in neurons may be involved in regulating neuronal mitochondrial density [14] as well as the cellular response to oxidative stress, and ROS can induce the PGC-1α-related expression of several genes coding for antioxidant enzymes [6]. Several molecules that contribute to the regulation of PGC-1α expression have been identified, including Creb [6], [15], [16], Forkhead Box, Class O (FOXO), Myocyte-Enhancer Factor 2 (MEF2), Peroxisome Proliferator-Activated Receptor and (PPAR, PPAR) and Estrogen Related Receptor (ERR) [17]–[19]. These activators of PGC-1α are variably distributed across tissues, and the specificity of activator tissue expression contributes to the specific regulation of PGC-1α [20]. Alternative splicing [21] and post-translational modifications [21], [22] provide further complexity to the regulation of PGC-1α activity.

PGC-1α has shown promise in animal models as a possible neuroprotective target in Huntington’s disease [23] and amyotrophic lateral sclerosis [24], [25]. In humans, variants in the *PGC-1α* gene have been found to possibly affect the age of onset of HD [26], [27], Alzheimer’s disease [28] and PD [29]. Mice lacking a functional copy of *Pgc-1α*, display neurodegenerative lesions in the striatum, and are substantially more sensitive to low doses of MPTP than wild-type littermates [6]. Furthermore, PGC-1α target genes are under-expressed in SN neurons at early stages of PD [30], implicating a role for low PGC-1α activity in PD. A mechanistic link between PD and PGC-1α comes from studies of Parkin, a gene in which loss of function mutations are associated with autosomal recessive early-onset PD [31], [32]. Parkin normally promotes degradation of PARIS, a protein that represses PGC-1α transcription, and therefore loss of Parkin function leads to reduced PGC-1α activity [33]. For these reasons we hypothesized that acute overexpression of Pgc-1α via a viral vector would protect against MPTP toxicity, and tested this hypothesis using an adeno-associated viral vector to overexpress full-length *Pgc-1α* in the SN of wild-type mice. In contrast to previously published work using transgenic overexpression of *Pgc-1α* [34] we found that AAV-mediated overexpression of *Pgc-1α* sensitized dopaminergic neurons to MPTP toxicity, and, in agreement with other work, reduced levels of striatal dopamine in a manner similar to that seen with AAV2/6 in female wild-type rats [35]. In addition, we found that loss of dopaminergic markers were associated with a loss of Pitx3, a key regulator of the dopaminergic phenotype and of the neurotrophic factor Bdnf.

## Methods

### Production of AAV Viral Vectors

The full length murine *Pgc-1α* gene (∼2.4 kb) was amplified by PCR using the flag-HA *Pgc-1α* pCDNA construct as the DNA template (generously provided by the Carles Lerin [36]). A SalI restriction site, Kozak sequence, and ATG start codon were added to the 5′ end of the *Pgc-1α* full length coding region using the forward primer 5′-GTTCTGGTCGACGCCACCATGGACTACAAGGACGACGATGAC-3′. A stop codon and HindIII restriction site were amplified at the 3′ end of the coding region using primer 5′-GTTCTGAAGCTTTCATCTCTGAGCTTCCTTCAGTAA-3′. The *Pgc-1α* PCR product was digested sequentially with SalI and HindIII and ligated into the same sites of the pACP-BHI plasmid (gift from Caroline Bass). The pACP-BHI plasmid contains the CMV promoter, a multiple cloning site and intron/polyA sequences derived from SV40. These elements are flanked by AAV2 inverted terminal repeats. The ligation was transformed into *Escherichia coli* DH5α using low temperature (30°C) growth and recovery of the DH5a to propagate the *FHA*-*Pgc-1α* vector. The final maxi-prep of the AAV-expression construct was sequenced to ensure correct insertion and the fidelity of the nucleotide sequence. Constructs consisting of EGFP-pACP and mCherry-pACP were used as controls. Packaging of the AAV2/10-*FHA*-*Pgc-1α,* AAV2/10-*mCherry* and AAV2/10-*EGFP* was carried out according to the standard triple transfection protocol to create helper virus-free pseudotyped AAV2/10 virus [37]. An AAV2/10 rep/cap plasmid provided the AAV2 replicase and AAV10 capsid genes [38]–[39], while the pHelper plasmid (Stratagene, La Jolla, CA) provided the adenoviral helper functions. Briefly, to produce virus, AAV- 293 cells grown in a 10 cm tissue culture dish were transfected with 10 µg of pHelper, 1.15 pmol each of AAV2/10 and AAV expression plasmids (*FHA*-*Pgc-1α-*pACP, mCherry-pACP or EGFP-pACP*)* via calcium phosphate precipitation. The cells were harvested 72 hours later, the pellets resuspended in DMEM, freeze-thawed and centrifuged several times to produced a clarified viral lysate. The virus was quantified using real-time PCR to measure the number of viral genome copies (vgc) contained within the intact virions.

### Intranigral AAV Injection

Adult (11–16 week) male C57BL/6 mice were obtained from Charles River Laboratories, Wilmington, MA. Mice were anesthetized with ketamine/xylazine and placed in a stereotaxic frame (myNeurolab, Leica Microsystems) with a mouse adapter. The tip of a pulled glass pipette (diameter) was inserted to stereotaxic coordinates AP:−2.9 mm; ML:−4.0 mm; DV:+1.2 mm, relative to bregma. Viral vector suspension in a volume of 1.5 µl was microinjected into the right hemisphere using 0.069 µl bursts from a Nanoliter 2000 injector (World Precision Instruments). This corresponded to 2×10^10^ viral genome copies (vgc) of AAV2/10-FHA-*Pgc-α* and 1×10^10^ vgc AAV2/10-*mCherry*.

### MPTP Treatment

1-Methyl-4-phenyl-1,2,3,6-tetrahydropyridine hydrochloride (MPTP; Sigma Aldrich) was re-suspended in 0.9% sterile saline (Hospia) to a final concentration of 3.3 mg/ml MPTP (calculated as free base). MPTP was administered via subcutaneous injection at a dose of 20 mg/kg body weight three weeks post-stereotaxic surgery. Injections were performed every two days for a period of 9 days (5 injections in total).

### Behavioral Analysis

Five days after the last MPTP or saline injection, mice were injected with 2 mg/kg body weight of amphetamine sulfate (Sigma Aldrich) in 0.9% sterile saline. After 15 minutes the mice were placed individually in a 28 (radius) × 37 (height) cm circular cylinder and behavior was recorded for 30 minutes after which time the mouse was returned to the home cage. The video recordings were scored by an individual blinded to treatment status, according to the number of ipsiversive or contraversive turns made, with one turn being defined as one full rotation of a point on the animal’s head past a marked point on the cylinder. Net scores were obtained by subtracting the number of contraversive turns from the number of ipsiversive turns.

### Ethics Statement

All animal procedures, as described above, were approved by the Beth Israel Deaconess Medical Center Animal Care and Use Committee (Permit numbers: 035-2008 and 027-2011).

### Immunofluorescent Staining Procedure

Two days after amphetamine-induced rotational behavior testing, mice were sacrificed via anesthetic overdose (ketamine/xylazine) and perfused with ice-cold 0.9% saline followed by 4% paraformaldehyde (PFA; Boston Bioproducts). Fixed brains were stored at 4°C in 4% PFA until cryopreservation for sectioning. Brains were cryoprotected by dehydration in 15% sucrose in PBS for 24 hours followed by 30% sucrose in PBS for 24 hours. Following cryoprotection, each brain was sectioned through the striatum, midbrain and SN at a thickness of 40 µm on a freezing microtome. Sections were permeabilized in TBS containing 0.25% Triton-X-100 for 30 minutes. The tissue sections were blocked in TBS +3% bovine serum albumin (BSA) for 1.5 hours at room temperature then incubated overnight at 4°C with the primary antibodies sheep anti-TH (AbCam) and rat anti-HA (Roche) each diluted 1∶1000 in TBS +1% BSA. The secondary antibodies Alexa Fluor donkey anti-sheep 594 and Alexa Fluor goat anti-rat 488 (Molecular Probes, Invitrogen) were diluted 1∶1000 in the same diluent and incubated with the tissue for 1.5 hours at room temperature. Sections were mounted with Vectashield mounting medium with DAPI (Vector Laboratories).

### Immunohistochemical Staining Procedures

Immunohistochemical staining for tyrosine hydroxylase (Th) was performed using a mouse monoclonal anti-Th antibody (Sigma Aldrich 1∶1000) and a Mouse-on-mouse kit (Vector Laboratories, Burlingame, CA) according to the manufacturer’s instructions. Immunohistochemistry for the dopamine transporter (Dat) was performed using a rat anti-DAT (Millipore) primary and a goat anti-rat IgG biotin-conjugated secondary antibody and a standard immunostaining protocol. Briefly, free-floating brain sections were permeabilized with 0.25% Triton-X-100 in TBS for 10 minutes and blocked in 3% normal goat serum (NGS, Jackson Immunolabs) in TBS for 30 minutes. All antibody incubations were performed using 1% NGS as a diluent. The sections were incubated with a 1∶5000 dilution of primary antibody overnight at 4°C and with secondary antibody diluted at 1∶1000 for 45 minutes. The ABC kit (Vector Laboratories) was used for signal amplification and binding of the antibody was visualized using diaminobenzidine with nickel intensification (Vector Laboratories). The sections were mounted on Superfrost Plus slides (VWR) and dehydrated through an ethanol series before clarifying with xylene and cover-slipping with Permaslip (Alban Scientific). A subset of samples were counterstained by immersion in thionin solution (pH 4.2–4.4) for 5 minutes before subsequent dehydration, clarification and cover-slipping.

### Quantitation of Immunostaining Intensity

For striatal densitometry, the stained sections were imaged using a light microscope fitted with a camera (SPOT) and images were captured using a 10× objective and fixed exposure settings. Densitometry was performed to quantify the intensity of Th and DAT immunostaining in the striatum ipsilateral to the microinjected SN and in the contralateral striatum. Specifically, using Adobe Photoshop, a region of interest 200×200 pixels was delineated in the striatum. The integrated density of this region was calculated using the software. Next, a 200×200 pixel region of interest was delineated over the adjacent cortex of the same section and the integrated density of this area calculated by the software. To normalize for differences in background staining intensity the cortical integrated density measurement was subtracted from the striatal integrated density measurement for each section analyzed.

### Stereological Neuronal Counts

For stereology, the midbrain of each mouse was sectioned using a freezing microtome into four series of 40 µm coronal sections and one series was stained with an anti-Th antibody, as before, for stereological cell counts in the SN. Stereology was performed using the optical fractionator method by an investigator blinded to treatment status. After mounting, each section in the series was observed using a 2.5 × objective lens and an outline was drawn around each SN and overlaid with a 50×50 counting frame using Stereo Investigator software (MicroBrightField). The nuclei of each Th+ cell within the boundaries of the optical fractionator (X 120, Y 120, height 10 µm; with 2 µm guard zones) was counted. The total numbers of Th+ cells per SN were then calculated by the optical fractionator software. A subset of samples were analyzed for total SN neuronal counts. In this case, stereology was performed in the same manner with a second marker used for simultaneous counts of thionin+/Th- neurons with Th- neurons being defined as those cells morphologically resembling neurons and with a visible nucleolus but lacking Th immunoreactivity.

### Measurement of DA and DA Metabolites

Mice were sacrificed by anesthetic overdose seven days after the final MPTP or saline injection. Animals used for these biochemical measurements had not undergone amphetamine-induced rotational behavior testing. The brains were rapidly removed, placed into a chilled brain matrix and sliced into 1 mm thick coronal sections on ice. The sections were then placed into ice-cold saline. The striatum was dissected from these 1 mm sections, snap-frozen and used for HPLC analysis of DA and DA metabolites, performed by the Neurochemistry Core, School of Medicine, Vanderbilt University. The levels of DA and DA metabolites were normalized to mg of protein input. Dopamine turnover was calculated as follows: DA turnover  =  (HVA + DOPAC)/DA.

### Western Blot Analysis

Brain tissue samples were homogenized in ice-cold lysis buffer using the TissueLyser LT with 5 mm stainless steel beads (Qiagen). Tissue was homogenized in 50 mM Tris-HCl (pH 7.4), 150 mM NaCl and 1% Triton-X 100 supplemented with protease inhibitors to a final concentration of 1× (Sigma-Aldrich). Protein concentrations were determined by Bicinchoninic Acid (BCA) Assay (Thermo Scientific) according to the manufacturer's instructions. Proteins were separated by SDS-PAGE on an appropriate Tris-HCl gel according to standard protocols. The membranes were blocked with 5% non-fat milk in either 1× PBS-0.1%Tween or 1× TBS-0.1%Tween. Primary antibodies were diluted in blocking solution in the following manner: mouse anti-β-actin (C4) (sc-47778, Santa Cruz) 1∶1000; mouse anti-Th (T2928, Sigma Aldrich) 1∶1000; rabbit anti-cyrochrome c oxidase subunit 4 (CoxIV) 1∶1000 (4850, Cell Signaling Technologies); mouse anti-Pgc-1α 1∶1000 (ST1202, Calbiochem) and incubated on the membranes with rocking overnight at 4°C. HRP conjugated anti-mouse IgG-HRP or anti-rabbit IgG (Santa Cruz and Cell Signaling Technology) were diluted 1∶1000 in blocking buffer and incubated with the membranes at room temperature for 1 hour. The LumiGOLD ECL Western Blotting Detection Kit (SignaGen) was used according to manufacturer's instructions and the blots were imaged by exposure on Amersham Hyperfilm ECL (GE Healthcare). Band densities were quantified using Adobe Photoshop as before [40]. Briefly, the image was converted to grayscale and inverted with respect to black and white (so that positive values correspond to darker bands) and the freehand selection tool was used to outline each band. Photoshop automatically computed the integrated density (the product of area and mean gray value) of each band. The integrated density of each band of interest was normalized to the integrated density for the loading control of that band.

### Expression Analysis

After dissection as before, RNA was extracted from the striata or SN using the RNeasy mini kit (Qiagen). After DNase treatment the RNA was purified using the RNeasy MinElute Clean-up kit (Qiagen) and used as a template for cDNA synthesis with High-Capacity cDNA Reverse Transcription Kit (Applied Biosystems). The final cDNA reaction was diluted seven-fold and 5 ul of the reaction was used in duplicate 15 ul SYBR-Green (Applied Biosystems) PCR reactions with primers specific to the target gene. Cycle numbers were normalized to the numbers obtained for a parallel 18S reaction, and fold-induction of the gene of interest was calculated in relation to the uninjected striatal or SN hemisphere of the same animal by the ΔΔCt method.

### Statistical Analyses

All statistical analyses were performed in Prism (Graphpad Software, La Jolla). For all comparisons of the microinjected hemisphere versus the contralateral hemisphere data were analyzed using a Student’s paired *t*-test. For comparisons between mCherry and Pgc-1α overexpressing SNs, the fold-changes relative to the contralateral sides were calculated and these values compared using Student’s *t*-test. Statistical comparisons of three groups or more were performed using a one-way ANOVA with *post hoc* Dunn’s Multiple Comparison Test. Numbers of mice indicated in the results section refer to numbers for each set of experimental conditions.

## Results

### AAV2/10 Expresses Functional Pgc-1α in the Nigro-striatal System

**Figure 1:**
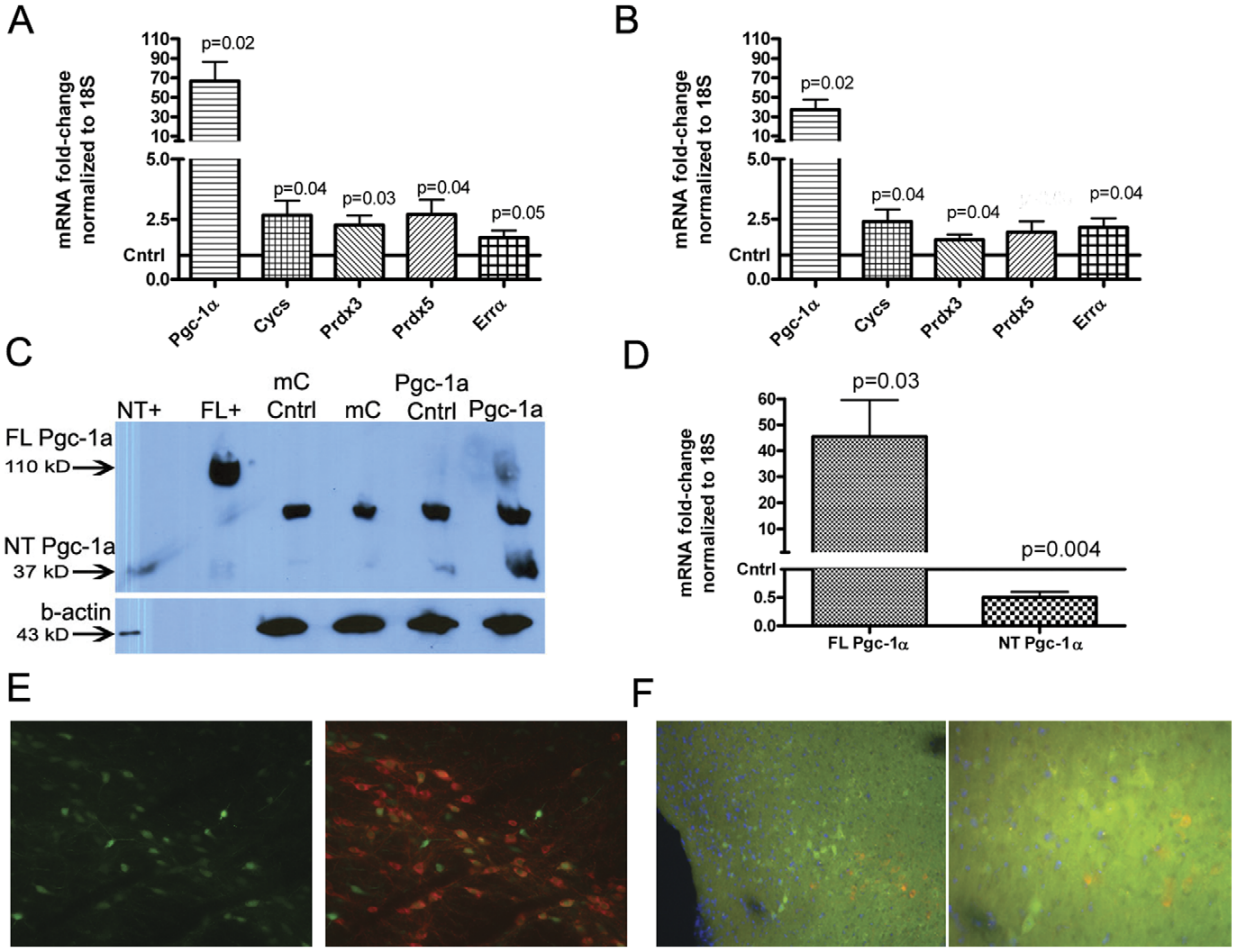
AAV2/10 expresses functional Pgc-1α in the nigro-striatal system. A. SYBR-green PCR analysis of *Pgc-1α* and *Pgc-1α*-target gene mRNA levels after saline treatment in mice microinjected unilaterally into the right SN with AAV2/10-FHA-*Pgc-1α* or AAV2/10-*mCherry*. mRNA levels were normalized to 18S and fold-change gene expression was calculated relative to the contralateral SN using the ΔΔCt method. Data were analyzed using a paired *t*-test (n = 6). **B.** The same analysis was performed after MPTP treatment (n = 6). **C.** Western blot analysis of pooled SN samples. 40 µg of protein was run on a 4–20% SDS-PAGE gel and the blot was probed with anti-Pgc1α antibody. Lanes 1 & 2 are positive controls for the 37-kD and 120 kD (full-length; FL) Pgc-1α isoforms respectively. Lanes 3 & 4 contain pooled whole-protein lysates from 3 contralateral SNs and mCherry-overexpressing (ipsilateral) SNs respectively. Lanes 5 and 6 lanes contain lysates from 3 contralateral SNs and Pgc-1α-overexpressing SNs respectively. **D.** SYBR-green PCR analysis of a separate set of SN samples for the mRNAs giving rise to the 120 kD (FL) and 35–38 kD (N-terminal; NT) *Pgc-1α* isoforms (n = 6). **E.** The left panel shows native EGFP fluorescence after injection of AAV2/10-EGFP into the SN. The right panel is a merged image of native EGPF (green) fluorescence and Th immunostaining (red). EGFP expressing Th+ cells appear orange/yellow. **F.** Immunofluorescence staining for the HA-tag (green), Th (red) and DAPI (blue; Magnification: × 40). The right panel shows the same immunofluorescently stained section at higher magnification (Magnification: × 200). G. Immunofluorescence staining of the same section shown in F shown here at higher magnification (Magnification: × 400). Panels from left to right: anti-HA, anti-TH, DAPI, merged image.

The AAV2/10-FHA-*Pgc-1α* vector was microinjected into the right-hemisphere SN of wild-type C57BL/6CR mice. The contralateral SN received no injection and served as control tissue. 1.5 µl of AAV2/10-FHA-*Pgc-1α,* corresponding to approximately 2×10^10^ vgc, or total intact virions injected, resulted in a mean 66.6-fold increase in *Pgc-1α* expression in the microinjected SN compared to the contralateral side (95% CI 15.73–117.6; p = 0.02) measured 5 weeks after injection (Figure 1A) by SYBR-green PCR using a primer set that does not discriminate between known *Pgc-1α* isoforms. This increase in *Pgc-1α* expression also induced target gene expression above baseline levels measured from the contralateral SN. Mean *Prdx3* expression increased 2.3-fold (95% CI 1.24–3.3; p = 0.03). A 2.7-fold increase in expression was observed for both *Prdx5* (95% CI 1.13–4.27; p = 0.04) and *Cycs* (95% CI 1.12–4.22; p = 0.04). There was a borderline significant 1.7-fold increase in mean *Errα* mRNA levels (95% CI 0.99 to 2.49; p = 0.05). AAV2/10-mCherry control injections did not significantly alter levels of expression of *Pgc-1α* or any of its target genes. In mice treated with 20 mg/kg MPTP (Figure 1B), the same titer of AAV2/10-FHA-*Pgc-1α* virus resulted in a 37.2-fold mean increase in *Pgc-1α* expression relative to the contralateral side (95% CI 11.03–63.45; p = 0.02). Again, Pgc-1α target genes were induced: *Errα* mRNA levels increased by a mean value of 2.15 (95% CI 1.09–3.12; p = 0.04), *Prdx3* expression increased 1.6-fold (95% CI 1.07 to 2.21; p = 0.04), and *Cycs* increased 2.4-fold (95% CI 1.08–3.71; p = 0.04). A trend towards increased expression of *Prdx5* after *Pgc-1α* overexpression and MPTP treatment did not reach statistical significance (95% CI 0.83 to 3.1; p = 0.08).

The 120 kD Pgc-1α protein was detected in the pooled samples from *Pgc-1α-*microinjected SNs (Figure 1C). We also detected a strong 35–38 kDa band that corresponded to the size of the published NT-Pgc-1α isoform [41] in the lysate from the Pgc-1α microinjected SN and in the contralateral SN of the same animals. This band was present only at a very low level in the ipsilateral and contralateral SNs of mCherry mice. This may indicate that some smaller Pgc-1α fragment may cross the midline into the contralateral hemisphere, or that unilateral Pgc-1α expression in one SN may have positive effects on Pgc-1α expression in the contralateral SN. However, analysis of *Pgc-1α* mRNA levels in the contralateral SN of Pgc-1α overexpressing animals compared to the contralateral SN of mCherry overexpressing animals, using the same cDNA samples as for Figure 1D, did not detect an increase in full-length *Pgc-1α* mRNA in the contralateral SN from ‘crossed over’ AAV2/10-FHA-*Pgc-1α* at time of sacrifice (data not shown).

The strong 35–38 kDa band seen on the western blot (Figure 1C) after *Pgc-1α* overexpression is unlikely to correspond to the published NT-Pgc-1α [41] isoform since the cDNA construct used lacks the intronic splice site required to generate this isoform. To investigate whether or not overexpressed full-length *Pgc-1α* could be inducing expression of an endogenous, shorter *Pgc-1α* transcript we measured *Pgc-1α* expression using primers specific for a shorter *Pgc-1α* isoform (a kind gift from Jorge Ruas, Figure 1D). Data were analyzed using a one-sample Student’s *t*-test with a theoretical mean of 1, corresponding to the baseline level of gene expression on the contralateral side, and we detected a 0.5-fold downregulation of this shorter transcript in the presence of AAV2/10-FHA-*Pgc-1α* compared to the contralateral side (95% CI 0.25 to 0.76; p = 0.004). Thus, the identity of the 35–38 kDa band seen on the western blot after *Pgc-1α* overexpression remains unknown. It is possible that this band represents misfolded protein, or a breakdown product of Pgc-1α that is not degraded as effectively as full-length Pgc-1α and retains the Pgc-1α epitope detected by the antibody. In addition, while this manuscript was in preparation several brain-specific Pgc-1α isoforms originating from an alternate promoter were identified [21]. Currently the expression-level of these novel isoforms in the SN remain undetermined.

A control AAV2/10-*egfp* virus spread well throughout the SN and was capable of transducing Th+ cells, as observed by viewing native fluorescence of EGFP (Figure 1E, green) on brain sections immunostained for Th (Figure 1E, red). Immunofluorescence staining for the HA-tag of the AAV2/10-FHA-*Pgc-1α* construct revealed expression of *Pgc-1α* in the SN (Figure 1F, green). However, surprisingly, the majority of HA-*Pgc-1a* was not co-localized with the expected pattern of Th immunofluorescence (Figure 1F, red). In addition, higher magnification (Figure 1F, right panel and figure 1G) revealed that the overexpressed Pgc-1α protein (green) was both nuclear (blue, DAPI fluorescence) and cytoplasmic.

### Overexpression of *Pgc-1α* in the SN Increases Mitochondrial Load in MPTP-treated Mice as Measured by Western Blot for CoxIV

**Figure 2:**
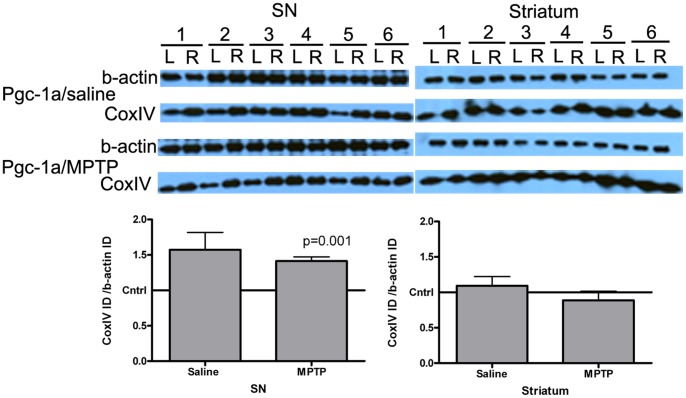
Western blot data for the mitochondrial marker CoxIV. 5 µg of whole-cell lysate from SN or striatal samples from saline or MPTP-treated Pgc-1α-microinjected mice was run on a 4–20% gradient gel and probed with anti-CoxIV and anti-b-actin antibodies. Each pair of lanes represents the contralateral (L) and ipsilateral (microinjected, R) SN respectively. Band densitometry data were analyzed using a paired Student’s *t*-test (n = 6 per group).

To determine whether *Pgc-1α* overexpression in the SN affected mitochondrial load, western blot for CoxIV was performed on whole-cell SN lysates (Figure 2). It can be seen that overexpression of *Pgc-1α* did not significantly alter CoxIV levels in the SN of saline-treated mice compared to the contralateral side, although there was a trend towards an increase (mean increase of 1.6-fold; 95% CI 0.94 to 2.20 p = 0.07). However, there was a significant 1.4-fold increase in CoxIV protein in the *Pgc-1α* overexpressing SN of MPTP-treated mice, relative to the contralateral side, regardless of whether the CoxIV band densitometry data was normalized to b-actin (95% CI 1.26 to 1.57; p = 0.001) or Tubb3 (95% CI 1.08 to 1.71; p = 0.024; n = 6, data not shown). There was no significant difference (0.35±0.33) between CoxIV band densitometry data when Pgc-1α microinjected SN samples from saline-treated or MPTP-treated animals were compared (95% CI −1.078 to 0.377; p = 0.308). A similar immunoblot using striatal tissue from *Pgc-1α*-microinjected animals did not detect increased CoxIV protein in the striatum ipsilateral to *Pgc-1α* overexpression (Figure 2, right-side blot).

### Unilateral Overexpression of *Pgc-1α* in the SN Leads to Ipsiversive Amphetamine-induced Rotational Behavior

**Figure 3:**
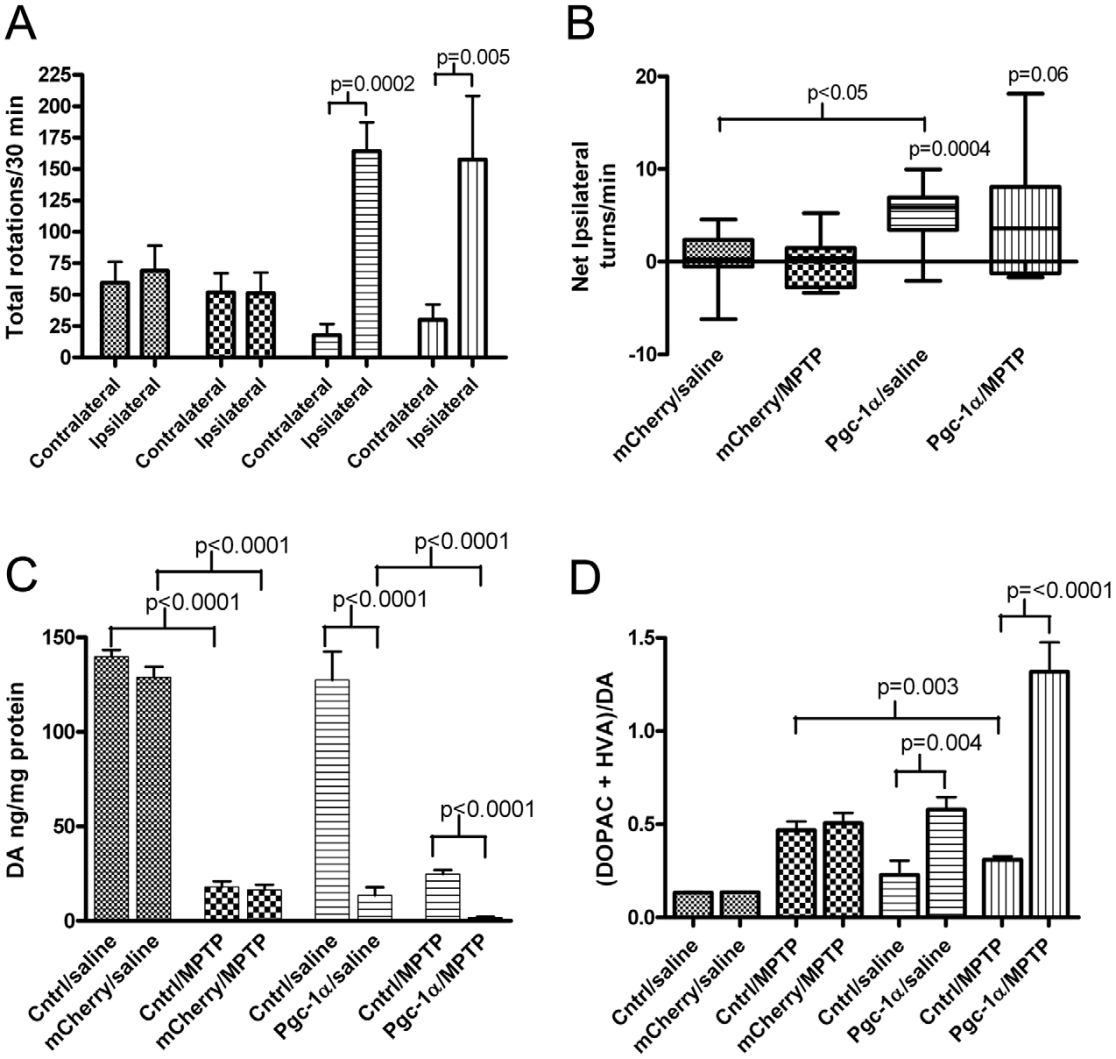
Amphetamine-induced rotational behavior and HPLC analysis of striatal DA. A. Mean number of ipsilateral and contralateral turns made by each group over the 30-minute test period following an IP injection of 2 mg/kg amphetamine. The data for average number of ipsilateral and contralateral turns made per group were compared using a 2-tailed t-test (mCherry/saline n = 12, mCherry/MPTP n = 9, Pgc-1α/saline n = 12, Pgc-1α/MPTP n = 10). All relevant statistically significant comparisons are indicated on the graph. **B.** Box and whiskers plot of net ipsilateral turns per minute per group averaged across each group. Data were analyzed by one-way ANOVA, all relevant statistically significant comparisons are shown on the graph. **C.** Mean levels of striatal DA per mg of protein in the ipsilateral and contralateral (cntrl) striatum of each treatment group. Data were analyzed by paired t-test. (mCherry/saline n = 14, mCherry/MPTP n = 15, Pgc-1α/saline n = 15, Pgc-1α/MPTP n = 16) **D.** DA turnover in each treatment group. Data were analyzed by paired t-test for within group comparisons and Student’s t-test for between group comparisons.

Mice were unilaterally microinjected in the (right) SN with AAV2/10-FHA-*Pgc-1α* or with AAV2/10-*mCherry* and 5 weeks later were exposed to MPTP or saline. Five days after the last saline or MPTP injection, the mice were assessed for rotational behavior induced by intraperitoneal administration of 2 mg/kg amphetamine (Figure 3A). Amphetamine tends to induce ipsiversive rotations in a mouse with a unilateral DA deficit. For this experiment, we predicted that unilateral overexpression of Pgc-1α would protect against MPTP toxicity ipsilateral to the injection, resulting in rotatations contraversive to the microinjected side. As expected, animals unilaterally overexpressing *mCherry* in the SN did not show a preferred direction of left-right rotation after amphetamine administration, as measured by net ipsilateral rotations/minute (Figure 3B), regardless of whether they were saline treated (0.32 net right rotations/minute; 95% CI −1.47 to 2.10; p = 0.70) or MPTP treated (−0.02 net right rotations/minute; 95% CI −2.15 to 2.11; p = 0.98). However, unexpectedly, mice that unilaterally overexpressed *Pgc-1α* in the SN exhibited a strong rotational preference toward the right-side, i.e. ipsiversive to *Pgc-1α* overexpression, regardless of whether the mice had received saline (mean 4.9 net right turns/minute; 95% CI 2.70 to 7.05; p = 0.0004) or MPTP treatment (mean 4.25 net right turns/minute; 95% CI −1.7 to 8.67; p = 0.06), although the p-value for the MPTP-treated mice was borderline. Also, the tendency to turn towards the right side in saline treated mice was significantly greater when the AAV2/10-FHA-*Pgc-1α* microinjected mice were compared to AAV2/10-*mCherry* microinjected mice (95% CI 1.91 to 7.21; p = 0.002). A similar tendency for mice microinjected with AAV2/10-FHA-*Pgc-1α* and treated with MPTP to turn towards the ipilateral side again did not quite reach statistical significance when analyzed by comparing AAV2/10-FHA-*Pgc-1α* to AAV2/10-*mCherry* microinjected mice (95% CI −9.0 to 0.46; p = 0.07), potentially due to statistical noise introduced by varying levels of MPTP toxicity between animals. These data indicate that unilateral overexpression of *Pgc-1α* leads to amphetamine-induced ipsilateral rotational behavior, suggesting that overexpression of *Pgc-1α* leads to DA depletion.

### Overexpression of Pgc-1α Reduces Striatal Levels of DA and DA Metabolites in Mouse Striatum

Stereotaxic injection of the AAV2/10-*mCherry* virus into the SN did not significantly alter levels of DA or DA metabolites (Figure 3C) as indicated by an absence of significant side-to-side differences in striata of these animals regardless of whether the animals were subsequently treated with saline (95% CI 0.85 to 1.01; p = 0.07) or MPTP (95% CI 0.84 to 1.03; p = 0.14).

In agreement with the ispiversive amphetamine-induced turning behavior, mice unilaterally overexpressing *Pgc-1α* in the SN have dramatically lower levels of DA and DA metabolites in the striatum ipislateral to *Pgc-1α* overexpression compared to the ipsilateral striatum of *mCherry* overexpressing mice. The mean DA level after *Pgc-1α* overexpression was 13.5 ng/mg protein, whereas in the mCherry animals this was 129.0 ng/mg protein, a difference of 115.4 ng/mg (95% CI 101.3 to 129.6; p = <0.0001). The mean level of DOPAC is reduced to 1.3 ng/mg protein after *Pgc-1α* overexpression compared to a mean level of 7.7 ng/mg protein after *mCherry* expression. For HVA the mean level is reduced to 3.3 ng/mg protein after *Pgc-1α* overexpression compared to 9.9 ng/mg protein after *mCherry* overexpression, and for 3-MT *Pgc-1α* overexpression yields a mean value of 2.8 ng/mg protein compared to 11.7 ng/mg protein for *mCherry*. Each of these comparisons is significant at the p = <0.0001 level. In addition, SN overexpression of *Pgc-1α* leads to significantly increased DA turnover from a mean ratio of 0.2 in the contralateral striatum to 0.58 in the ipsilateral striatum, an increase of 0.36 (Figure 3D; 95% CI 0.13 to 0.57; p = 0.004), representing a mean 2.9-fold increase. This effect is further pronounced after treatment with MPTP where DA turnover is increased by 1.0 (95% CI 0.660 to 1.356; p = <0.0001) from a mean ratio of 0.31 to 1.32 after *Pgc-1α* overexpression, representing a mean 4.3-fold increase.

### Overexpression of *Pgc-1α* in the SN Leads to a Loss of Striatal Th Immunoreactivity

**Figure 4:**
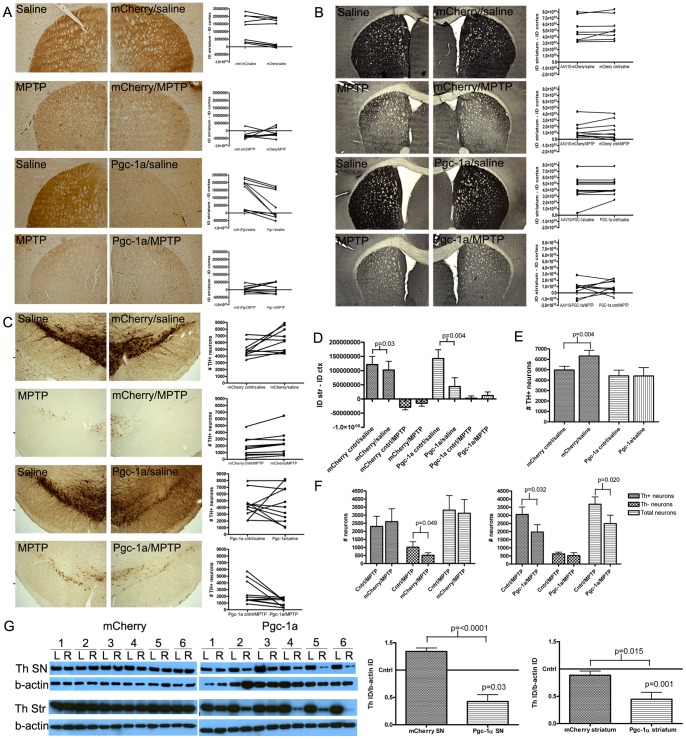
Th and Dat immunohistochemical staining and stereological cell counts. A. Representative images of the striatum used for Th densitometry with line graphs showing the integrated density of Th immunoreactivity for each animal analyzed. In the line graphs, for each animal a line connects the results for the uninjected side (contralateral, cntrl) and the microinjected side (mCherry or PGC-1a) (mCherry/saline n = 9, mCherry/MPTP n = 8, Pgc-1α/saline n = 9, Pgc-1α/MPTP n = 8). **B.** Representative images of the striatum used for Dat densitometry with line graphs showing the integrated density of Dat immunoreactivity for each animal analyzed (mCherry/saline n = 8, mCherry/MPTP n = 10, Pgc-1α/saline n = 11, Pgc-1α/MPTP n = 10). **C.** Representative images of the SN used for Th+ cell stereological analysis with line graphs showing the number of Th+ cells for each animal analyzed (mCherry/saline n = 10, mCherry/MPTP n = 8, Pgc-1α/saline n = 11, Pgc-1α/MPTP n = 10). **D.** Striatal Th immunoreactivity data for all treatment groups. Ipsilateral and contralateral striatal Th immunoreactivity within each treatment group was analyzed using a paired Student’s *t*-test (mCherry/saline n = 9, mCherry/MPTP n = 8, Pgc-1α/saline n = 9, Pgc-1α/MPTP n = 8). All significant comparisons are indicated on the graph. **E.** SN Th+ stereological cell count data for all treatment groups. The number of Th+ cells in the ipsilateral vs contralateral SNs within each treatment group were analyzed using a paired t-test. (mCherry/saline n = 10, Pgc-1α/saline n = 11). All significant comparisons are indicated on the graph. **F.** SN stereology data from MPTP-treated animals (mCherry and Pgc-1α) showing the number of Th+ neurons, Th- neurons (thionin staining) and total SN neurons (combined data). Data were analyzed using paired t-tests to compare the contralateral and ipsilateral (microinjected) SNs in each treatment group and Student’s t-test for comparing between groups. (mCherry/MPTP n = 8, Pgc-1α/MPTP n = 10). **G.** Western blot analysis of Th immunoreactivity in gross-dissected whole cell lysate SN and striatal samples (saline groups only). Band densitometry data was analyzed using a paired t-test to compare Th-band intensity between the contralateral and ipsilateral (microinjected) SN and striatum (n = 6 per group).

Overexpression of *Pgc-1α* in the SN caused an average 69.4% reduction in Th immunoreactivity, a mean reduction of 9.9×10^7^ AU (95% CI 4.2×10^7^ to 16×10^7^; p = 0.004; Figure 4A), from a mean adjusted integrated density of 14×10^7^ AU in the contralateral striatum compared to 4.4×10^7^ AU in the striatum ipsilateral to microinjection. Overexpression of *mCherry* in the SN also significantly reduced Th immunoreactivity in the striatum from an average adjusted integrated density of 12×10^7^ AU to 10×10^7^ AU, a mean reduction of 1.9×10^7^ AU (95% CI 0.29×10^7^ to 3.5×10^7^; p = 0.03), a much lesser decrease than that seen following *Pgc-1α* overexpression. These data indicate that the injection procedure, the virus itself, or the mCherry protein had a mild impact on Th immunostaining in the striatum, but this impact accounts for less than 20% of the magnitude of effect seen following *Pgc-1α* overexpression. Evidence that *Pgc-1α* overexpression induces a reduction in levels of Th protein, beyond that seen with mCherry is further demonstrated by western blot data (Figure 4G) where there is no loss of Th protein in mCherry SN or striatal samples.

As expected, MPTP treatment largely ablated Th expression in the striatum (Figures 4A and 4D) and this loss of Th immunoreactivity was not exacerbated by overexpression of either *mCherry* (95% CI −2.1×10^7^ to 5.0×10^7^; p = 0.38) or *Pgc-1α* (95% CI −1.4×10^7^ to 3.4×10^7^; p = 0.38), potentially reflecting a floor effect.

Immunoreactivity for the DA transporter (Dat; Figure 4B) was not significantly altered by *mCherry* overexpression with a mean difference of 2.7×10^7^±9.3×10^7^ between striatal hemispheres (95% CI −2.3×10^8^ to 1.7×10^8^; p = 0.77) or *Pgc-1α* with a mean difference of 2.2×10^7^±7.0×10^7^ (95% CI −1.7×10^8^ to 1.2×10^8^; p = 0.75) prior to MPTP treatment, indicating no significant loss of dopaminergic terminals after overexpression of either gene.

Stereological cell counts of Th+ neurons in the SN (Figures 4C, 4E and 4F) revealed an increased average number of Th+ neurons counted on the AAV2/10-*mCherry* expressing SN compared to the contralateral SN in saline-treated animals (Figure 4E). From an average 4978 Th+ cells per SN to an average 6323 Th+ cells per SN, a mean difference of 1345 Th+ cells (95% CI 93.9 to 2595; p = 0.038). In contrast, *Pgc-1α* overexpression did not affect the number of Th+ cells in the SN of saline-treated animals. The mean number of Th+ cells was comparable between the contralateral side (4124 Th+ cells) and the *Pgc-1α* overexpressing side (4170 Th+ cells; 95% CI −1820 to 1795; p = 1.0). However, as ascertained from total cell counts, *Pgc-1α* overexpression caused a significant decrease in Th+ cell survival following MPTP treatment (Figure 4F), from a mean 3057 Th+ cells on the contralateral side to 1975 Th+ cells on the *Pgc-1α* overexpressing side, a mean difference of 1082 TH+ cells (95% CI 117 to 2047; p = 0.03). In addition, we found that Pgc-1α overexpression did not significantly alter the number of thionin+/Th- neurons (628 contralateral, 521 ipsilateral, mean difference 108; 95% CI −294.1 to 509.5; p = 0.56). Therefore, the significant difference in mean total cell counts between the contralateral SN (3686 neurons) and the Pgc-1α overexpressing SN (2496 neurons, mean difference 1190; 95% CI 232.8 to 2146; p = 0.02) after MPTP treatment was attributed predominantly to increased Th+ cell death in response to MPTP treatment. This increased loss of Th+ cells was not observed in mCherry microinjected SNs after MPTP.

### Overexpression of *Pgc-1α* in the SN Decreases Expression of Dopaminergic Cell Markers

Loss of Th at the protein level after *Pgc-1α* overexpression was confirmed by western blot using whole-cell lysates derived from gross dissected SN tissue samples (Figure 4G). By this measure mean Th levels were reduced by 57.5% following *Pgc-1α* overexpression in the SN ipsilateral to the injection compared to the contralateral side in the saline treated animals, a difference of 0.9054 AU (95% CI 0.487 to 1.324; p = 0.03) and by 76.8% in the MPTP treated mice, a difference of 0.935 AU (95% CI 0.432 to 1.44; p = 0.005; data not shown). In contrast, no reduction in Th protein levels was observed by western blot using whole-cell lysates derived from gross dissected ipsilateral SN tissue samples from mCherry overexpressors compared to the contralateral side. A similar pattern of results was also seen for striatal samples (Figure 4G). Quantitative SYBR-green PCR was performed using gene-specific primers and cDNA derived from gross dissected microinjected-SN and contralateral SN tissue (data not shown). Relative mean expression of *Th* was reduced to 0.125 (95% CI 0.124 to 0.238; p = <0.0001; n = 6), *Dat* was reduced to 0.08 (95% CI 0.02 to 0.142; p = <0.0001; n = 5) and *Vmat* was reduced to 0.146 (95% CI 0.056 to 0.237; p = <0.0001; n = 5) in *Pgc-1a* overexpressing SN tissue compared to a defined mean of 1 for the contralateral SN.

**Figure 5:**
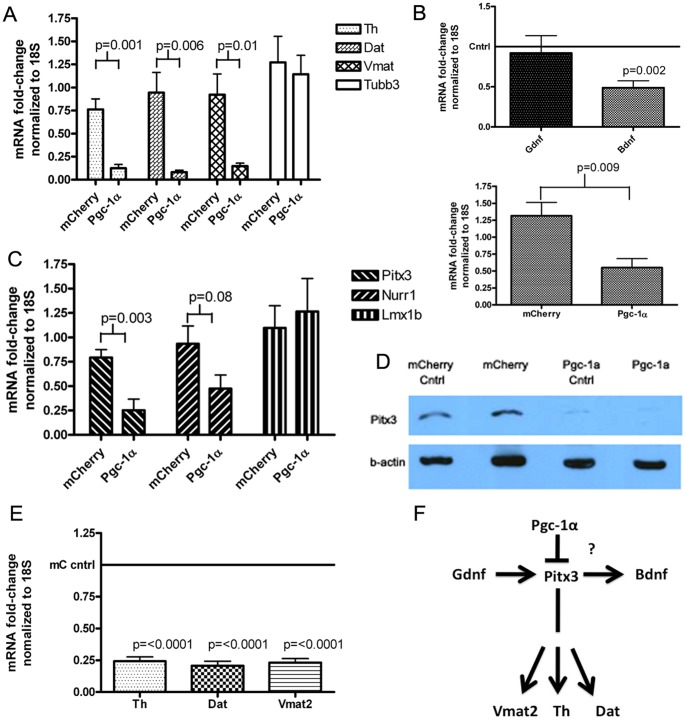
Analysis of markers of the dopaminergic phenotype. A. SYBR-green PCR analysis of mRNA levels of *Th, Dat* and *Vmat2* in mCherry and Pgc-1α overexpressing SN samples (mCherry n = 6, Pgc-1α n = 5). **B.** Upper panel. SYBR-green PCR analysis of mRNA levels of *Gdnf* and *Bdnf* in Pgc-1α SN samples. Fold-change in gene expression was calculated with respect to the contralateral SN using the ΔΔCt method with normalization to 18S. Data were analyzed using a one-sample t-test to determine if fold-change differed from the baseline value of 1 (n = 6 per group). Lower panel. Bdnf expression in mCherry and Pgc-1α overexpressing SN samples. Fold-change was calculated as before and the data were compared using Student’s t-test (n = 6 each group). **C.** SYBR-green PCR analysis of mRNA levels of *Pitx3, Nurr1* and *Lmx1b* in mCherry and Pgc-1α overexpressing SN samples. Fold-change in gene expression relative to the contralateral side was calculated as before and data were analyzed by comparing the mean fold change between groups using a Student’s t-test (mCherry n = 6, Pgc-1α n = 5, except for *Lmx1b* where the analysis was performed on a separate set of samples; mCherry n = 6, Pgc-1α n = 4). **D.** Western blot analysis of Pitx3 protein levels in pooled SN whole-cell lysate (same samples as 1D). “Cntrl” refers to the contralateral control side. **E.** SYBR-green PCR analysis of mRNA levels of *Th, Dat* and *Vmat2* in the contralateral (cntrl) SN from mCherry and Pgc-1α overexpressing mice. Fold-change gene expression was calculated in relation to the overall mean gene expression level for the contralateral SN of all mCherry overexpressing animals (n = 6 for both groups). Data were analyzed using a one-sample *t*-test to determine if fold-change gene expression differed from 1. **F.** Schematic showing convergence of the genes found to be down-regulated after *Pgc-1α* overexpression, on Pitx3.

These markers of the dopaminergic phenotype were also reduced in the SN when the mean fold-change in gene expression (again, calculated for the side ipsilateral to microinjection relative to the contralateral) for *Pgc-1α* overexpressing mice was compared to that for *mCherry* overexpressing mice (Figure 5A). Again, the effects were striking; *Th* expression was reduced after Pgc-1α overexpression to a mean level of 0.125±0.004 compared to 0.76±0.113 after mCherry, a mean difference of 0.6±0.13 (95% CI 0.34 to 0.93; p = 0.0008). *Dat* expression was reduced to 0.08±0.02 after Pgc-1α overexpression compared to 0.94±0.22 after *mCherry* overexpression a difference of 0.86±0.24 (95% CI 0.31 to 1.413; p = 0.006). *Vmat2* expression was also reduced from 0.92±0.2 after *mCherry* overexpression to 0.146±0.03 after *Pgc-1α* expression a difference of 0.775±0.25 (95% CI 0.21 to 1.3; p = 0.013).

### Over-expression of *Pgc-1α* in the SN Decreases Expression of the Neurotrophic Factor *Bdnf*

To ascertain possible reasons for increased sensitivity to MPTP-induced reduction in Th+ cells in the *Pgc-1α* over-expressing SN of MPTP-treated mice, SYBR-green quantitative PCR was performed using the SNs of Pgc-1α-microinjected animals to determine the mRNA level of the growth factors *Gdnf* and *Bdnf* (Figure 5B). Although the level of *Gdnf* was unchanged between the contralateral and *Pgc-1α-*microinjected SN, *Bdnf* mRNA levels were significantly decreased in the *Pgc-1α* overexpressing SNs compared to the contralateral striatum in the saline-treated condition by a mean difference of 0.51 (95% CI 0.29 to 0.74; p = 0.002), and when comparing the mean side-to-side differences for the *Pgc-1α* versus *mCherry* groups where the mean of the differences was 0.77±0.24 (95% CI 0.24 to 1.3; p = 0.009). The *Pgc-1α* dependent decrease in *Bdnf* expression in the *Pgc-1α* overexpressing SN versus the contralateral SN is not apparent after MPTP treatment (data not shown), which may represent upregulation of *Bdnf* after MPTP treatment or, preferential loss of the cells with lower levels of *Bdnf* expression on the *Pgc-1α* overexpressing side.

### Expression of the Transcription Factor *Pitx3* is Reduced by *Pgc-1α* Over-expression in the SN

*Th*, *Dat*, *Vmat2* and *Bdnf* are all downstream of the transcription factor Pitx3, whereas GDNF is upstream [42]. Pitx3 can also work in concert with another transcription factor, Nurr1, to facilitate expression of the dopaminergic phenotype [43]. SYBR-green quantitative PCR revealed that over-expression of *Pgc-1α* resulted in a significant down-regulation of *Pitx3* to 0.25±0.11 from a 0.79±0.08 level of expression after mCherry (Figure 5C, 95% CI 0.23 to 0.85; p = 0.003), a nonsignificant trend toward lower *Nurr1* expression (0.47±0.14 from 0.93±0.18, a difference of 0.46±0.24; 95% CI −0.08 to 1.0; p = 0.08), and no loss of *Lmx1b* expression relative to *mCherry* microinjected SN (1.26±0.33 from 1.1±0.23, a difference of −0.17±0.39; 95% CI −1.07 to 0.73; p = 0.677). Other transcription factors and co-activators (*Atf4*, *Mta-1* and *Dj-1*) were not affected (data not shown), indicating that the effect of Pgc-1α on *Pitx3* does not reflect a nonspecific global suppression of transcription. We also confirmed a loss of Pitx3 at the protein level (Figure 5D). Consistent with some crossing over of Pgc-1α protein, and loss of Th, Vmat2 and Dat mRNA expression in the contralateral SN (Figure 1E), this loss of Pitx3 protein was observed to a lesser extent in the contralateral SN in addition to the Pgc-1α overexpressing SN. Given the large loss of Pitx3 at the protein level, it may be expected that we would see effects on Pitx3 target-gene expression in the contralateral SN. *Post-hoc* analysis of dopaminergic marker gene expression data from the contralateral (uninjected) SN, demonstrated that Pitx3 target genes (Th, Dat and Vmat2) were indeed downregulated at the mRNA level in the contralateral SN of Pgc-1α overexpressing animals compared to the contralateral SN of mCherry overexpressing animals (Figure 5E; p<0.0001 in all cases).

### Lower Levels of *Pgc-1α* Viral Over-expression also Disrupt the Dopaminergic Phenotype

**Figure 6:**
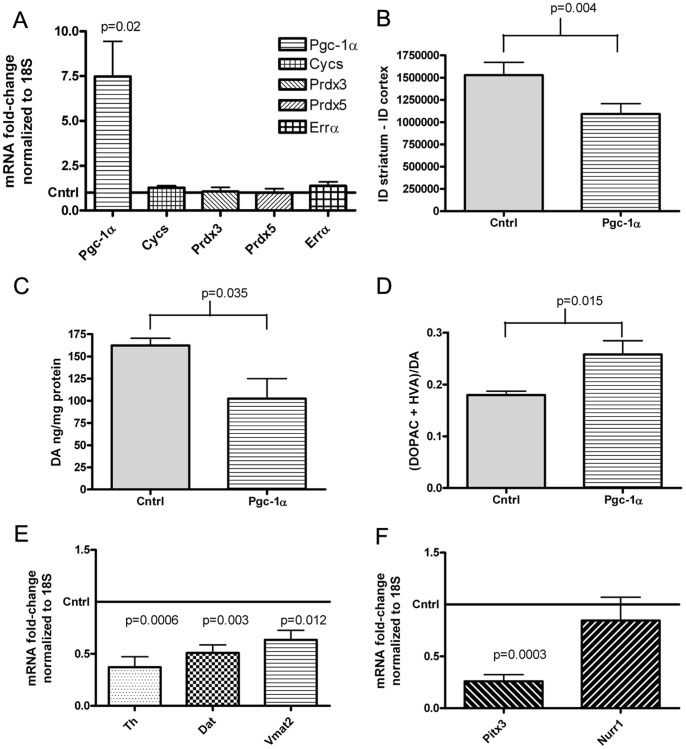
Data from lower-titer injections. A. SYBR-green PCR analysis of Pgc-1α and Pgc-1α target gene mRNA levels after saline treatment in mice microinjected unilaterally into the right SN with AAV2/10-FHA-Pgc-1α or AAV2/10-mCherry. mRNA levels were normalized to 18S and fold-change gene expression was calculated relative to the contralateral SN using the ΔΔCt method. Data were analyzed using a paired *t*-test (n = 6). **B.** Striatal Th immunoreactivity data for the contralateral side (Cntrl) compared to the ipsilateral side microinjected in the SN with AAV2/10-FHA-PGC-1α. Ipsilateral and contralateral striatal Th immunoreactivity within each treatment group was analyzed using a paired t-test (n = 7). **C.** Mean levels of striatal DA per mg of protein in the ipsilateral and contralateral (cntrl) striatum of mice microinjected unilaterally with a lower titer of AAV2/10-FHA-PGC-1α. Data were analyzed by paired t-test (n = 5). **D.** DA turnover in each treatment group. Data were analyzed by paired t-test for within group comparisons and Student’s t-test for between group comparisons. **E.** SYBR-green PCR analysis of dopaminergic markers. Fold change in gene expression relative to the contralateral side was calculated as before. Data were analyzed using a one-sample t-test to determine if fold-change gene expression differed from 1 (n = 5). **F.** SYBR-green PCR analysis of *Pitx3* and *Nurr1* expression (n = 5). Fold change was calculated and data were analyzed as in E.

To determine whether the effects observed in the SN after *Pgc-1α* over-expression could be linked to the level of *Pgc-1α* over-expression, a lower titer of the Pgc-1α vector, 1×10^9^ vgc in a 1.5 µl volume, was microinjected into the right SN of wild-type C57BL/6CR mice (Figure 6A). This resulted in a mean 6.48-fold increase in *Pgc-1α* expression (95% CI 1.4 to 11.54; p = 0.02). With this lower titer injection, the impact of Pgc-1α on the expression of the subset of Pgc-1α-target genes examined was no longer detectible. This may reflect a low sensitivity for detecting small changes in gene expression levels since mRNA levels from tissue homogenate were examined, which likely includes RNA from many cells that were not transduced.

Although the effects were less pronounced, a significant 28.6% loss of Th immunoreactivity in the striatum was observed (Figure 6B) from 1.53×10^6^ AU on the contralateral side to 1.09×10^6^ on the ipsilateral side, a difference of 4.38×10^5^ AU (95% CI 2.0×10^5^ to 6.7×10^5^; p = 0.004), as was loss of DA, from an average of 162 ng/mg protein to 102 ng/mg protein a difference of 60.02 ng/mg protein (Figure 6C, 95% CI 6.034 to 114.0; p = 0.035). DA turnover in the striatum (Figure 6D) also increased from 0.18±0.01 to 0.258±0.002, an increase of 0.078 (95% CI −0.1378 to −0.018). Lesser in magnitude, but still significant effects also were seen for downregulation of *Th* (Figure 6E) to a mean expression level of 0.37 (95% CI 0.09 to 0.65; p = 0.003), *Dat* to a mean expression level of 0.51 (95% CI 0.28 to 0.71; p = 0.003), *Vmat2* to a mean expression level of 0.64 (95% 0.11 to 0.62; p = 0.02) and *Pitx3* to a mean expression level of 0.26 (95% CI 0.57 to 0.92; p = 0.0003) mRNA levels in the ipsilateral SN compared to the contralateral side. These data are consistent with suppression of the dopaminergic phenotype by Pgc-1α even with these lower titer injections.

## Discussion

PGC-1α is a transcriptional co-activator that can induce mitochondrial biogenesis and upregulate antioxidant defenses [44] [6], [22]. This, along with the finding of low expression of PGC-1α target genes in SN neurons in early PD [30], has led to the suggestion that increasing PGC-1α activity may be neuroprotective in Parkinson’s disease. However, data presented here indicate that there may be unintended and deleterious effects of viral overexpression of Pgc-1α in the SN.

We achieved a robust level of *Pgc-1α* expression at the mRNA level, and increased expression of the Pgc-1α-target genes *Prdx3*, *Prdx5* and *Cycs* (with high titer injections) coupled with an increase in the mitochondrial marker CoxIV in the SN after MPTP treatment, indicating that the overexpressed Pgc-1α was functional. We also detected *Pgc-1α* protein expression; albeit low levels of the full-length Pgc-1α isoform, which may be expected for a transcriptional co-activator with a short half-life [45], [46] [47].

Although our AAV2/10-EGFP virus spread well throughout the SN and was capable of transducing Th+ cells, we had difficulty finding convincing co-localization of Pgc-1α and Th in neurons after microinjection of AAV2/10-FHA-Pgc-1α. While this was initially surprising, the likely reason for this observation (downregulation of *Th* expression by *Pgc-1α*) became clear after further investigation.

Another unexpected finding was that we observed no evidence of neuroprotection after *Pgc-1α* overexpression, and in fact, observed enhanced vulnerability to MPTP as evidenced by reduced numbers of total neurons by stereological cell counts in the SN. This was particularly unexpected since *Pgc-1α* knockout animals are more sensitive to MPTP toxicity [6], and other recent work suggests that constitutive transgenic overexpression of *Pgc-1α* from the *Thy1* promoter may be protective against MPTP toxicity [34]. In addition, we observed substantial depletion of dopamine (independent of MPTP toxicity) as a result of *Pgc-1α* overexpression in the SN, a potentially deleterious effect if considering PGC-1α as a neuroprotective strategy for PD. Together, these data raise the possibility that there may be a narrow window of optimal activity of PGC-1α, whereby either too little or too much is detrimental, a conclusion that is consistent with the precise regulation of PGC-1α activity that normally occurs.

The observed deleterious effects may reflect the high levels of Pgc-1α activity achieved via viral vector microinjection. Unfortunately, quantification of the level of Pgc-1α mRNA or protein has not been reported in many of the published overexpression studies, making it difficult to compare levels of expression between studies. In addition, in the case of transgenic overexpression of *Pgc-1α,* compensatory changes associated with overexpression throughout development could contribute to the fact that transgenic mice were protected against MPTP toxicity, whereas we detect increased sensitivity to MPTP with viral *Pgc-1α* overexpression in adult wild-type mice.

While this manuscript was in preparation another article also identified deleterious effects resulting from viral overexpression of *PGC-1α* in the nigro-striatal system [35]. These authors used an AAV2/6 viral vector to induce a 400-fold increase in *PGC-1α* mRNA in rat SN, and observed Th+ neuronal loss in the absence of any other treatment. In our study, AAV2/10 mediated *Pgc-1α* overexpression induced loss of dopaminergic markers without inducing overt neuronal death, suggesting that additional toxicities may become apparent at higher levels of overexpression. Consistent with this, these authors observed only a ‘minimal impact’ on survival of Th+ SN neurons with lower-levels of *PGC-1α* expression in the SN (achieved with injections in the striatum rather than directly into the SN), further indicating that the level of *PGC-1α* overexpression may be crucial. The time-period post *Pgc-1α* expression may also be an important factor in observing Th+ cell death. In the previously published study, rats were sacrificed 3 months after *PGC-1α* overexpression, considerably longer than our 7-week interval in mice.

In the current study, levels of *Pgc-1α* mRNA expression at time of sacrifice ranged from a 13-fold increase to a 122-fold increase relative to the contralateral side for the higher titer injections and from 1.6-fold to 13-fold for the lower titer injections. Since mRNA levels were measured from gross dissected SN tissue, and not every cell in the SN was transduced by the virus, it is likely that the level of expression in individual transduced cells was much higher than the average fold-change values obtained, and that such high levels of expression may have deleterious effects that outweigh any potential protective actions.

There is a precedent for adverse effects reported in association with PGC-1α overexpression, particularly in muscle tissues [48], [49], which may be linked to linked to excessive proliferation of mitochondria. In our study, levels of the mitochondrial marker CoxIV were not increased in the AAV2/10-FHA-*Pgc-1α* microinjected SN at baseline (saline treatment) relative to the contralateral SN, and although the levels of CoxIV were increased in the AAV2/10-FHA-*Pgc-1α* microinjected SN relative to the contralateral SN after MPTP-treatment, there was no statistically significant difference between CoxIV band intensity in the Pgc-1α microinjected SNs when comparing SNs of saline versus MPTP treated mice. Therefore, the increase in CoxIV levels in the MPTP-treated mice likely represents maintenance of CoxIV levels as a result of *Pgc-1α* expression in the presence of MPTP.

Despite maintaining CoxIV levels, we observed an increased loss of striatal DA, and Th staining, in the *Pgc-1α* overexpressing mice after MPTP treatment, compared to saline treatment. This, in conjunction with lower total SN neuronal counts, indicates an increased sensitivity to MPTP at this level of *Pgc-1α* overexpression. Potentially, this could be linked to *Bdnf* downregulation at baseline. BDNF is an important neurotrophic factor for dopaminergic neurons [50], [51], is reduced in the SN in PD [52], [53]and many studies have shown that overexpression of *Bdnf* is protective in experimentally-induced PD models [50], [54]–[56]. In addition, it may be that Bdnf and Th act synergistically in enhancing dopaminergic cell survival in response to a neurotoxic insult [57], a point that is particularly relevant to this work given the observed downregulation of Th in response to Pgc-1α overexpression.

Recent work has shown that *Bdnf* is a downstream target of Gdnf and Pitx3 [42], a transcription factor involved in the development and maintenance of dopaminergic neurons [58]–[60]. Pitx3 also mediates the expression of *Th*, *Vmat2* and *Dat* [61]–[62] [63], [64], which we found to be strongly downregulated in response to *Pgc-1α* overexpression, both in the presence and absence of MPTP administration. Therefore, we hypothesized, and subsequently confirmed, that viral overexpression of *Pgc-1α* downregulates *Pitx3* expression in the SN. The downregulation of *Pitx3* provides a link between *Bdnf*, *Th*, *Vmat2*, *Dat* mRNA downregulation in this system (Figure 5F). Consistent with this, levels of *Gdnf,* which is upstream of *Pitx3*, were not altered by *Pgc-1α*. In light of the recent finding by Peng et al [42] that Pitx3 mediates Gdnf-induced upregulation of Bdnf, it will be interesting to determine how many of the effects on Th cells historically attributed to Gdnf are mediated by the effect of Gdnf on Pitx3 and Bdnf.

Given the well-established key role of Pitx3 in Th expression, which is required for DA synthesis, it is likely that downregulation of Pitx3 contributes to the DA depletion that we observed following unilateral overexpression of Pgc-1α. However, Bdnf may be involved in this as Bdnf has been implicated in the positive regulation of Th expression during development [65].

Several lines of data demonstrate that downregulation of *Pitx3* and Pitx3 target gene expression is not reflective of a nonspecific global downregulation of transcription. Levels of Tubb3, a neuronal marker, were not altered by *Pgc-1α* expression. Also, other mRNAs were found to be unaltered by Pgc-1α, including levels of *Gdnf*, as well as expression of several other transcription factors and co-activators (*Atf4*, *Lmx1b*, *Mta-1* and *Dj-1*).

Th was downregulated at both the mRNA level and at the protein level, as measured by qPCR, immunohistochemistry and western blot. In contrast, Dat was only downregulated at the mRNA level, whereas Dat immunostaining in the striatum was similar between *mCherry* and *Pgc-1α* overexpressing animals. The disparity between *Dat* mRNA and protein levels may be due to temporal factors; i.e. although transcription of *Dat* is reduced, the degradation of membrane-spanning and membrane associated Dat protein by the endosomal/lysosomal pathway [66] has not yet proceeded to a level where the reduced replenishment of Dat protein is detectable. In contrast cytosolic Th, which is noticeably and quantifiably lost at the protein level in this system, is primarily degraded in a more rapid manner by the proteasome [67]–[68].

This work demonstrates that viral vector mediated overexpression of full-length *Pgc-1α* in the SN does not protect against MPTP-induced neuronal loss and DA depletion. Indeed, viral overexpression of *Pgc-1α* potentiated MPTP toxicity in Th+ neurons. Our data further show that Pgc-1α induces loss of the dopaminergic phenotype in SN neurons, potentially due, to reduced *Pitx3* expression. While Pgc-1α induced DA depletion is an important concern for the use of full-length Pgc-1α gene therapy as a neuroprotective in PD, it is unlikely to have direct ramifications for *Pgc-1α* overexpression strategies in other areas of the brain since the expression of *Pitx3* is localized to midbrain neurons. Although Pitx3 suppression likely contributes to the downregulation of the dopaminergic phenotype associated with *Pgc-1α* overexpression, further studies are needed to elucidate the mechanism by which *Pgc-1α* downregulates *Pitx3*. PGC-1α remains in theory a promising target for neuroprotection, but careful attention to the precise levels of PGC-1α expression and the method of Pgc-1α modulation [69] will likely be necessary in order to achieve the protective potential of PGC-1α while avoiding its potentially deleterious effects.

## References

[1] Simon DK, MF Beal (2001) Pathogenesis: oxidative stress, mitochondrial dysfunction and excitotoxicity, in Parkinson's Disease: Diagnosis and Clinical Management, S.A. Factor and W.J. Weiner, Editors. Demos Vermande: New York. p. chapt 27.

[2] Beal MF (2003) Mitochondria, oxidative damage, and inflammation in Parkinson's disease. Ann N Y Acad Sci 991: p. 120–31. 10.1111/j.1749-6632.2003.tb07470.x 12846981

[3] Schapira AH, Gegg M (2011) Mitochondrial Contribution to Parkinson's Disease Pathogenesis. Parkinson's Disease, 2011. 10.4061/2011/159160 PMC3109314 21687805

[4] Jenner P (2003) *Oxidative stress in Parkinson's disease.* Ann Neurol. 53 Suppl 3: p. S26–36; discussion S36–8. 10.1002/ana.10483 12666096

[5] Lin MT, MF Beal (2006) Mitochondrial dysfunction and oxidative stress in neurodegenerative diseases. Nature. 443(7113): p. 787–95. 10.1038/nature05292 17051205

[6] St-Pierre J, Drori S, Uldry M, Silvaggi JM, Rhee J, et al.. (2006) Suppression of reactive oxygen species and neurodegeneration by the PGC-1 transcriptional coactivators. Cell. 127(2): p. 397–408. 10.1016/j.cell.2006.09.024 17055439

[7] Valle I, Alvarez-Barrientos A, Arza E, Lamas S, Monsalve M, et al. (2005) *PGC-1{alpha} regulates the mitochondrial antioxidant defense system in vascular endothelial cells.* Cardiovasc Res. 66(3): p. 562–573. 10.1016/j.cardiores.2005.01.026 15914121

[8] Wullner U, Loschmann P-A, Schultz JB, Schmid A, Dringen R, et al. (1996) *Glutathione depletion potentiates MPTP and MPP+ toxicity in nigral dopaminergic neurones.* Neuroreport. 7(4): p. 921–3. 10.1097/00001756-199603220-00018 8724674

[9] Esterbauer H, Oberkofler H, Krempler F, Patsch W (1999) Human peroxisome proliferator activated receptor gamma coactivator 1 (PPARGC1) gene: cDNA sequence, genomic organization, chromosomal localization, and tissue expression. Genomics 62(1): p. 98–102. 10.1006/geno.1999.5977 10585775

[10] Knutti D, A Kaul, Kralli A (2000) A tissue-specific coactivator of steroid receptors, identified in a functional genetic screen. Mol Cell Biol. 20(7): p. 2411–22. 10.1128/mcb.20.7.2411-2422.2000 PMC85422 10713165

[11] Baar K, Wende AR, Jones TE, Marison M. Nolte LA, et al.. (2002) Adaptations of skeletal muscle to exercise: rapid increase in the transcriptional activator PGC-1. Faseb J. 16(14): p. 1879–86. 10.1096/fj.02-0367com 12468452

[12] Pilegaard H, Saltin B, Neufer PD (2003) Exercise induces transient transcriptional activation of the PGC-1alpha gene in human skeletal muscle. J Physiol. 546: p. 851–8. 10.1113/jphysiol.2002.034850 PMC2342594 12563009

[13] Puigserver P, Wu Z, Park C-W, Graves R, Wright M, et al. (1998) *A cold-inducible coactivator of nuclear receptors linked to adaptive thermogenesis.* Cell 92(6): p. 829–39. 10.1016/s0092-8674(00)81410-5 9529258

[14] Wareski P, Vaarmaan A, Choubey V, Safiulina D, Liiv J, et al. (2009) *PGC-1{alpha} and PGC-1{beta} regulate mitochondrial density in neurons.* J Biol Chem. 284(32): p. 21379–85. 10.1074/jbc.M109.018911 PMC2755862 19542216

[15] Handschin C, Li P, Liu F, Maratos-Flier E, et al.. (2007) Skeletal muscle fiber-type switching, exercise intolerance, and myopathy in PGC-1alpha muscle-specific knock-out animals. J Biol Chem. 282(41): p. 30014–21. 10.1074/jbc.M704817200 17702743

[16] Herzig S, Long F, Jhala US, Hedrick S, Quinn R, et al. (2001) *CREB regulates hepatic gluconeogenesis through the coactivator PGC-1.* Nature. 413(6852): p. 179–83. 10.1038/35093131 11557984

[17] Czubryt MP, McAnally J, Fishman GI, Olson EN (2003) Regulation of peroxisome proliferator-activated receptor gamma coactivator 1 alpha (PGC-1 alpha ) and mitochondrial function by MEF2 and HDAC5. Proc Natl Acad Sci U S A 100(4): p. 1711–6. 10.1073/pnas.0337639100 PMC149898 12578979

[18] Handschin C, Spiegelman BM (2006) Peroxisome proliferator-activated receptor gamma coactivator 1 coactivators, energy homeostasis, and metabolism. Endocr Rev. 27(7): p. 728–35. 10.1210/er.2006-0037 17018837

[19] Wang L, Liu J, Saha P, Huang J, Chan L, et al.. (2005) The orphan nuclear receptor SHP regulates PGC-1alpha expression and energy production in brown adipocytes. Cell Metab. 2(4): p. 227–38. 10.1016/j.cmet.2005.08.010 16213225

[20] Lin J, Handschin C, Spiegelman BM (2005) *Metabolic control through the PGC-1 family of transcription coactivators.* Cell Metab. 1(6): p. 361–70. 10.1016/j.cmet.2005.05.004 16054085

[21] Soyal SM, Felder TK, Auer S, Hahne P, Oberkofler H, et al.. (2012) A greatly extended PPARGC1A genomic locus encodes several new brain-specific isoforms and influences Huntington disease age of onset. Hum Mol Genet. 21(15): p. 3461–3473. 10.1093/hmg/dds177 22589246

[22] Clark J, Simon DK (2008) Transcribe to Survive: transcriptional control of antioxidant defense programs for neuroprotection in Parkinson's disease. Antioxid Redox Signal, 2008. 10.1089/ars.2008.2241 18717631

[23] Cui L, Jeong H, Borovecki E, Parkhurst CN, Tanese N, et al.. (2006) Transcriptional Repression of PGC-1[alpha] by Mutant Huntingtin Leads to Mitochondrial Dysfunction and Neurodegeneration. Cell. 127(1): p. 59–69. 10.1016/j.cell.2006.09.015 17018277

[24] Zhao W, Varghese M, Yemul S, Pan Y, Cheng A, et al.. (2011) Peroxisome proliferator activator receptor gamma coactivator-1alpha (PGC-1alpha) improves motor performance and survival in a mouse model of amyotrophic lateral sclerosis. Mol Neurodegener. 6(1): p. 51. 10.1186/1750-1326-6-51 PMC3156746 21771318

[25] Liang H, Ward WF, Jang YC, Bhattacharya A, Bokov AF, et al.. (2011) PGC-1alpha protects neurons and alters disease progression in an amyotrophic lateral sclerosis mouse model. Muscle Nerve. 44(6): p. 947–56. 10.1002/mus.22217 22102466

[26] Taherzadeh-Fard E, Saft C, Andrich J, Wieczorek S, Arning L (2009) *PGC-1alpha as modifier of onset age in Huntington disease.* Molecular Neurodegeneration. 4(1): p. 10. 10.1186/1750-1326-4-10 PMC2644307 19200361

[27] Weydt P, Soyal S, Gellera C, DiDonato S, Weidinger C, et al. (2009) *The gene coding for PGC-1alpha modifies age at onset in Huntington's Disease.* Molecular Neurodegeneration. 4(1): p. 3. 10.1186/1750-1326-4-3 PMC2630305 19133136

[28] Helisalmi S, Vepsalainen S, Hiltunen M, Koivisto AM, Salminen A, et al. (2008) *Genetic study between SIRT1, PPARD, PGC-1alpha genes and Alzheimer's disease.* J Neurol. 255(5): p. 668–73. 10.1007/s00415-008-0774-1 18438697

[29] Clark J, Reddy S, Zheng K, Betensky RA, Simon DK (2011) Association of PGC-1alpha polymorphisms with age of onset and risk of Parkinson's disease. BMC Med Genet. 12: p. 69. 10.1186/1471-2350-12-69 PMC3112073 21595954

[30] Zheng B, Liao Z, Locascio JJ, Lesniak KA, Roderick SS (2010) *PGC-1alpha, a potential therapeutic target for early intervention in Parkinson's disease.* Sci Transl Med. 2(52): p. 52ra73. 10.1126/scitranslmed.3001059 PMC3129986 20926834

[31] Kitada T, Asakawa S, Hattori N, Matsumine H, Yamamura Y, et al. (1998) *Mutations in the parkin gene cause autosomal recessive juvenile parkinsonism.* Nature. 392(6676): p. 605–8. 10.1038/33416 9560156

[32] Lucking CB, Durr A, Bonifati V, Vaughan J, De Michele G, et al.. (2000) Association between early-onset Parkinson's disease and mutations in the parkin gene. French Parkinson's Disease Genetics Study Group. N Engl J Med. 342(21): p. 1560–7. 10.1056/NEJM200005253422103 10824074

[33] Shin JH, Ko HS, Kang H, Lee Y, Lee Y-I, et al.. (2011) PARIS (ZNF746) repression of PGC-1alpha contributes to neurodegeneration in Parkinson's disease. Cell. 144(5): p. 689–702. 10.1016/j.cell.2011.02.010 PMC3063894 21376232

[34] Mudo G, Di Liberto V, Tselykh TV, Oliveri M, et al.. (2012) Transgenic expression and activation of PGC-1alpha protect dopaminergic neurons in the MPTP mouse model of Parkinson's disease. Cell Mol Life Sci. 69(7): p. 1153–65. 10.1007/s00018-011-0850-z PMC11114858 21984601

[35] Ciron B, Lengacher S, Dusonchet J, Aebischer P, Schneider BL (2012) Sustained expression of PGC-1alpha in the rat nigrostriatal system selectively impairs dopaminergic function. Hum Mol Genet. 21(8): p. 1861–76. 10.1093/hmg/ddr618 PMC3313800 22246294

[36] Lerin C, Rodgers JT, Kalume DE, Kim SH, Pandey A, et al.. (2006) GCN5 acetyltransferase complex controls glucose metabolism through transcriptional repression of PGC-1[alpha]. Cell Metabolism. 3(6): p. 429–438. 10.1016/j.cmet.2006.04.013 16753578

[37] Xiao X, Li J, Samulski RJ (1998) Production of high-titer recombinant adeno-associated virus vectors in the absence of helper adenovirus. J Virol. 72(3): p. 2224–32. 10.1128/jvi.72.3.2224-2232.1998 PMC109519 9499080

[38] Gao GP, Alvira MR, Wang L, Calcedo R, Johnston J, et al. (2002) *Novel adeno-associated viruses from rhesus monkeys as vectors for human gene therapy.* Proc Natl Acad Sci U S A 99(18): p. 11854–9. 10.1073/pnas.182412299 PMC129358 12192090

[39] De BP, Heguy A, Heckett N, Ferris B, Leopold P, et al.. (2006) High levels of persistent expression of alpha1-antitrypsin mediated by the nonhuman primate serotype rh.10 adeno-associated virus despite preexisting immunity to common human adeno-associated viruses. Mol Ther. 13(1): p. 67–76. 10.1016/j.ymthe.2005.09.003 16260185

[40] Clark J, Clore EL, Zheng K, Adame A, Masliah E, et al.. (2010) Oral N-acetyl-cysteine attenuates loss of dopaminergic terminals in alpha-synuclein overexpressing mice. PLoS One. 5(8): p. e12333. 10.1371/journal.pone.0012333 PMC2925900 20808797

[41] Zhang Y, Huypens P, Adamson AW, Chang JS, Henagan TM, et al. (2009) *Alternative mRNA splicing produces a novel biologically active short isoform of PGC-1alpha.* J Biol Chem. 284(47): p. 32813–26. 10.1074/jbc.M109.037556 PMC2781698 19773550

[42] Peng C, Aron L, Klein R, Li M, Wurst W, et al.. (2011) Pitx3 is a critical mediator of GDNF-induced BDNF expression in nigrostriatal dopaminergic neurons. J Neurosci. 31(36): p. 12802–15. 10.1523/JNEUROSCI.0898-11.2011 PMC6623418 21900559

[43] Jacobs FM, van Erp S, van der Linden AJA, von Oerthel L, Burbach PH, et al. (2009) *Pitx3 potentiates Nurr1 in dopamine neuron terminal differentiation through release of SMRT-mediated repression.* Development. 136(4): p. 531–40. 10.1242/dev.029769 19144721

[44] Puigserver P, Spiegelman BM (2003) Peroxisome proliferator-activated receptor-gamma coactivator 1 alpha (PGC-1 alpha): transcriptional coactivator and metabolic regulator. Endocr Rev. 24(1): p. 78–90. 10.1210/er.2002-0012 12588810

[45] Puigserver P, Rhee J, Lin J, Wu Z, Yoon JC, et al.. (2001) Cytokine stimulation of energy expenditure through p38 MAP kinase activation of PPARgamma coactivator-1. Mol Cell. 8(5): p. 971–82. 10.1016/s1097-2765(01)00390-2 11741533

[46] Olson BL, Hock MB, Ekholm-Reed S, Wohlschlegel JA, Dev KK, et al.. (2008) SCFCdc4 acts antagonistically to the PGC-1alpha transcriptional coactivator by targeting it for ubiquitin-mediated proteolysis. Genes Dev. 22(2): p. 252–64. 10.1101/gad.1624208 PMC2192758 18198341

[47] Trausch-Azar J, Leone TC, Kelly DP, Schwartz AL (2010) Ubiquitin proteasome-dependent degradation of the transcriptional coactivator PGC-1{alpha} via the N-terminal pathway. J Biol Chem. 285(51): p. 40192–200. 10.1074/jbc.M110.131615 PMC3001001 20713359

[48] Russell LK, Mansfield CM, Lehman JJ, Kovacs A, Courtois M, et al.. (2004) Cardiac-specific induction of the transcriptional coactivator peroxisome proliferator-activated receptor gamma coactivator-1alpha promotes mitochondrial biogenesis and reversible cardiomyopathy in a developmental stage-dependent manner. Circ Res. 94(4): p. 525–33. 10.1161/01.RES.0000117088.36577.EB 14726475

[49] Miura S, Tomitsuke E, Yasutomi K, Yamazaki T, et al.. (2006) Overexpression of peroxisome proliferator-activated receptor gamma co-activator-1alpha leads to muscle atrophy with depletion of ATP. Am J Pathol. 169(4): p. 1129–39. 10.2353/ajpath.2006.060034 PMC1780180 17003473

[50] Hyman C, Hofer M, Barde Y-M, Juhasz M, Yancopoulos GD, et al. (1991) *BDNF is a neurotrophic factor for dopaminergic neurons of the substantia nigra.* Nature. 350(6315): p. 230–2. 10.1038/350230a0 2005978

[51] Loeliger MM, Briscoe T, Rees SM (2008) *BDNF Increases Survival of Retinal Dopaminergic Neurons after Prenatal Compromise.* Inv Ophthamol & Vis Sci. 49(3): p. 1282–1289. 10.1167/iovs.07-0521 18326759

[52] Parain K, Murer MG, Yan Q, Faucheux B, Agid Y, et al.. (1999) Reduced expression of brain-derived neurotrophic factor protein in Parkinson's disease substantia nigra. NeuroReport. 10(3): p. 557–561. 10.1097/00001756-199902250-00021 10208589

[53] Levivier M, Przedborski S, Bencsis C, Kang UJ (1995) Intrastriatal implantation of fibroblasts genetically engineered to produce brain-derived neurotrophic factor prevents degeneration of dopaminergic neurons in a rat model of Parkinson's disease. J Neurosci. 15(12): p. 7810–7820. 10.1523/JNEUROSCI.15-12-07810.1995 PMC6577965 8613721

[54] Frim DM, Uhler TA, Galpern WR, Beal MF, Breakefield XO, et al.. (1994) Implanted fibroblasts genetically engineered to produce brain-derived neurotrophic factor prevent 1-methyl-4-phenylpyridinium toxicity to dopaminergic neurons in the rat. Proc Natl Acad Sci U S A. 91(11): p. 5104–8. 10.1073/pnas.91.11.5104 PMC43940 8197193

[55] Galpern WR, Frim DM, Tatter SB, Altar CA, Beal MF, et al.. (1996) Cell-mediated delivery of brain-derived neurotrophic factor enhances dopamine levels in an MPP+ rat model of substantia nigra degeneration. Cell Transplant. 5(2): p. 225–32. 10.1177/096368979600500211 8689033

[56] Tsukahara T, Takeda M, Shimohama S, Ohara O, Hashimoto N (1995) Effects of Brain-derived Neurotrophic Factor on 1-Methyl-4-phenyl-1,2,3,6-tetrahydropyridine-induced Parkinsonism in Monkeys. Neurosurgery. 37(4): p. 733–741. 10.1227/00006123-199510000-00018 8559303

[57] Wang ZH, Ji Y, Shan W, Zeng B, Raksadawan N, et al.. (2002) Therapeutic effects of astrocytes expressing both tyrosine hydroxylase and brain-derived neurotrophic factor on a rat model of Parkinson's disease. Neuroscience. 113(3): p. 629–40. 10.1016/s0306-4522(02)00204-x 12150782

[58] van den Munckhof P, Luk KC, Ste-Marie, Montgomery J, Blanchet P, et al. (2003) *Pitx3 is required for motor activity and for survival of a subset of midbrain dopaminergic neurons.* Development. 130(11): p. 2535–42. 10.1242/dev.00464 12702666

[59] Smidt MP, Smits SM, Burbach JP (2004) Homeobox gene Pitx3 and its role in the development of dopamine neurons of the substantia nigra. Cell Tissue Res. 318(1): p. 35–43. 10.1007/s00441-004-0943-1 15300495

[60] Yang D, Peng C, Li X, Fan X, Li L, et al.. (2008) Pitx3-transfected astrocytes secrete brain-derived neurotrophic factor and glial cell line-derived neurotrophic factor and protect dopamine neurons in mesencephalon cultures. J Neurosci Res. 86(15): p. 3393–400. 10.1002/jnr.21774 18646205

[61] Lebel M, Gauthier Y, Moreau A, Drouin J (2001) Pitx3 activates mouse tyrosine hydroxylase promoter via a high-affinity binding site. J Neurochem. 77(2): p. 558–67. 10.1046/j.1471-4159.2001.00257.x 11299318

[62] Maxwell SL, Ho Y, Kuehner E, Zhao S, Lin M (2005) Pitx3 regulates tyrosine hydroxylase expression in the substantia nigra and identifies a subgroup of mesencephalic dopaminergic progenitor neurons during mouse development. Dev Biol. 282(2): p. 467–79. 10.1016/j.ydbio.2005.03.028 15950611

[63] Messmer K, Remington MP, Skidmore F, Fishman PS (2007) *Induction of tyrosine hydroxylase expression by the transcription factor Pitx3.* Int J Dev Neurosci. 25(1): p. 29–37. 10.1016/j.ijdevneu.2006.11.003 17184956

[64] Hwang DY, Hong DY, Jeong JW, Choi S, Kim H, et al.. (2009) Vesicular monoamine transporter 2 and dopamine transporter are molecular targets of Pitx3 in the ventral midbrain dopamine neurons. J Neurochem. 111(5): p. 1202–12. 10.1111/j.1471-4159.2009.06404.x PMC4896488 19780901

[65] Fukuchi M, Fujii H, Takachi H, Ichinose H, Kuwane Y, et al.. (2010) Activation of tyrosine hydroxylase (TH) gene transcription induced by brain-derived neurotrophic factor (BDNF) and its selective inhibition through Ca2+ signals evoked via the N-methyl-d-aspartate (NMDA) receptor. Brain Research. 1366(0): p. 18–26. 10.1016/j.brainres.2010.10.034 20965158

[66] Daniels GM, Amara SG (1999) Regulated trafficking of the human dopamine transporter. Clathrin-mediated internalization and lysosomal degradation in response to phorbol esters. J Biol Chem. 274(50): p. 35794–801. 10.1074/jbc.274.50.35794 10585462

[67] Doskeland AP, Flatmark T (2002) Ubiquitination of soluble and membrane-bound tyrosine hydroxylase and degradation of the soluble form. Eur J Biochem. 269(5): p. 1561–9. 10.1046/j.1432-1033.2002.02808.x 11874472

[68] Nakashima A, Mori K, Kaneko YS, Hayashi N, Nagatsu T, et al. (2011) *Phosphorylation of the N-terminal portion of tyrosine hydroxylase triggers proteasomal digestion of the enzyme.* Biochem Biophys Res Commun. 407(2): p. 343–7. 10.1016/j.bbrc.2011.03.020 21392500

[69] Lindholm D, Ericksson O, Makela J, Belluardo N, Korhonen L (2012) *PGC-1α: a master gene that is hard to master.* Cell Mol Life Sci. 69: p. 2465–2468. 10.1007/s00018-012-1043-0 PMC11114758 22678664

